# Supplementary material for: Are carbon emissions trading and green financial instruments synergistic? -Comprehensive quantitative research based on content analysis
Source: PLoS One. 2024 Mar 7;19(3):e0298601. doi: 10.1371/journal.pone.0298601 (PMC10919601; doi:10.1371/journal.pone.0298601)
Supplement: S1 Data — (ZIP) [file pone.0298601.s001.zip › Data/地级市Z_F工作报告.zip!/║■─╧/╒┼╝╥╜τ╩╨/└┤╫╘╣1⁄2╓┌║┼í╛┬φ┐╦╩2╛▌═°í┐_2014.docx]

各位代表：

　　现在，我代表市人民政府向大会作工作报告，请予审议，并请各位政协委员及其他列席人士提出意见。

　　一、2013年工作回顾

　　2013年是我市面临严重困难、经历严峻挑战的一年，也是我市攻坚克难、砥砺奋进的一年。一年来，面对国际国内宏观环境复杂、经济下行压力加大、1959年以来最严重干旱等多重不利因素，全市上下在省委、省政府和市委的坚强领导下，在市人大、市政协的支持监督下，科学把握形势变化，积极应对各种挑战，紧紧围绕&ldquo;提质张家界，打造升级版&rdquo;战略部署，组织实施&ldquo;双联双解六攻坚&rdquo;主题活动，全力以赴推进旅游升温、项目建设、企业生产、城市管理、招商引资、投资融资&ldquo;六大攻坚战&rdquo;，迎难而上，团结拼搏，稳增长、促转型、强基础、惠民生，心无旁骛抓发展，只争朝夕搞建设，保持了经济社会平稳较快发展。

　　&mdash;&mdash;经济运行逆势向好，经济发展稳中有进。全市实现生产总值365亿元（预计数，下同），同比增长6%，其中一、二、三次产业增加值分别增长3.2 %、10.5%和 4.7 %。完成固定资产投资205亿元，同比增长20%，其中实施重点工程项目43个，完成投资50.8亿元，同比增长17.6%。实现社会消费品零售总额126亿元，同比增长12%。实现财政总收入35.75亿元、公共财政预算收入25.13亿元，同比分别增长11%和11.8%。城镇居民人均可支配收入达到17000元、农村居民人均纯收入达到5070元，同比分别增长10%和11%。城镇登记失业率和居民消费价格涨幅分别控制在4%和3%以内。

　　&mdash;&mdash;项目建设势头强劲，投资拉动稳健有力。坚持基础先行、投资拉动，切实加快项目建设进程。重大交通项目建设实现突破。张花高速公路建成通车，荷花国际机场新航站楼破土动工，张桑高速公路、黔张常铁路建设蓄势待发，安慈、宜张、桑龙、张新等高速公路前期工作进一步夯实。兴建、续建干线公路110公里，共完成建设投资5亿元，同比增长8.4%。城乡基础设施项目建设快速推进。以&ldquo;五路&rdquo;改造为重点，中心城区大庸东路、大庸中路、大庸西路、鼎泰路、大桥路、子午西路二期提质改造工程如期全面竣工，华天城滨河路、阳光大道顺利建成。慈利、桑植县城新区建设取得实质性进展。全市新建和改造城乡电网1317公里，完成农村公路建设283公里、渡改桥及危桥改造58座，整修病险水库30座，治理河道38.3公里，新建、整修农田灌溉渠道212公里，实施农村土地整治6054公顷。产业项目建设步伐加快。碧桂园、禾田居、纳百利等高星级休闲度假酒店建成，晟昊国际旅游商业城基本竣工，华天城一期主体工程基本完成，杨家界索道、武陵源游客服务中心、张家界大峡谷二期工程、钢贸城、汽贸城等项目建设顺利推进。重大能源项目&mdash;&mdash;桑植、慈利页岩气开发正式启动勘探。民生和社会事业项目有力实施。市人民医院和张家界航院整体搬迁工程启动实施，市技工学校整体搬迁项目建设进度过半，市博物馆主体工程基本完成。建成义务教育合格学校28所，实施19个乡镇卫生院、72个村卫生室提质改造。新建各类保障性住房5580套，为10475个农户配置清洁能源，解决8.75万农村人口饮水安全问题。招商引资成效凸显。

出台招商引资管理办法，强化了招商引资决策机制、推进机制和激励机制。大力加强招商引资项目库建设，精心策划和包装重大招商项目32个。重点抓好&ldquo;港洽周&rdquo;、&ldquo;香港企业湘西行&rdquo;等重大招商，扎实开展台湾等一系列专题招商，取得丰硕成果。全年成功签约重大招商项目27个，概算投资规模达到183亿元；实际利用外资6100万美元，实际利用内资37亿元，同比分别增长14.8%和11.4%。要素保障进一步加强。金融机构贷款规模同比增长17%，净增信贷40亿元。全市共争取到位国家和省里投资64亿元，同比增长11%。新增建设用地418公顷，同比增长36%。

　　&mdash;&mdash;旅游升温强力实施，客源结构积极变化。针对今年以来旅游市场急剧下滑的严峻形势，及时果断采取措施，突重围，抢市场，打出了一系列旅游升温组合拳。大规模组织旅游促销。深入长三角、珠三角、京津唐、西南、西北、东北等主要客源地&ldquo;找问题、找市场、找客源&rdquo;，巩固了市场份额。举办了第3届国际乡村音乐周、第2届世界翼装飞行锦标赛、第17届国际森林保护节、法国蜘蛛侠徒手攀爬百龙天梯、国际网络摄影赛、全国滑水锦标赛、全国自行车穿越峰林等一系列重大营销活动，在国际国内引起普遍关注。深化与央视等强势媒体和腾讯等新兴媒体合作，拓展与英国《孤独星球》等境外媒体合作，成立&ldquo;重庆&middot;张家界旅游联盟&rdquo;，打造了一批宣传促销平台。完善出台旅游营销奖励办法，适时推出全球中小学生免门票、成年游客错峰旅游优惠等政策，形成了强劲的市场吸引力。旅游航线拓展取得突破。开通了张家界至台湾定期直航，实现了台湾游客落地签注，巩固和扩大了张家界至泰国包机直航，新增了张家界至合肥、珠海航线，台湾、东南亚、长三角、珠三角客源市场更加活跃，航空口岸入境旅客成倍增长，首破10万人大关。旅游环境质量进一步提升。以国家出台和实施《旅游法》为契机，切实深化&ldquo;平安满意在张家界&rdquo;专项活动，大力整治旅游市场环境，创建&ldquo;智慧旅游&rdquo;系统，推行旅游标准化服务，全市旅游的美誉度和满意度明显提升，被评为&ldquo;2013年中国旅游竞争力百强市&rdquo;。经过不懈努力，全市景点共接待游客3440万人次，其中过夜游客1425万人次，虽然同比分别下降了4.1%和4%，但旅游总收入达到212亿元，同比增长1.7%,自驾游、自助游和散客比重快速增长，旅游消费水平进一步提高。

　　&mdash;&mdash;城市管理不断加强，建设秩序明显改观。坚持&ldquo;在建设中管理、在管理中建设&rdquo;，围绕服务游客、服务市民，让游客满意、让市民满意的&ldquo;双服务、双满意&rdquo;目标，强力实施城市管理攻坚，取得阶段性成果。违法建设势头得到有效遏制。下大气力整治违法建设，形成了综合整治浓厚氛围。中心城区实现违法建设发现率、违法建设建档率、新的违法建设拆除整改到位率三个100%，全年共拆除新老违法建筑18万平方米。城镇规划体系建设不断加快。组织编制了且住岗、沙堤片区控制性详规，提出了澧水风貌带规划控制要点，市城区法定图则管理深入落实。县城、乡镇和村庄规划加快推进，城乡规划体系进一步完善。市政配套设施建设扎实推进。完成了澄潭水厂二期扩建工程，实施了排污干管、供水管网完善配套和改造提质工程，检修下水管道142公里，新建天然气管网25.6公里，新增城市绿地3.6万平方米，施划城市交通标示标线1.7万平方米，铺设人行道板2400平方米，清理确定停车泊位11797个。市容市貌明显好转。市城区日常保洁、垃圾清运处理机制全面建立，开展&ldquo;道路大清洗、渣土大整治、垃圾大扫除&rdquo;专项行动，街面卫生质量大幅提高，大力消除小街小巷环卫盲点，摊点、噪音和视觉污染治理全面铺开，&ldquo;门前三包&rdquo;取得实效，卫生城市、文明城市、交通管理模范城市创建工作逐步深化。

　　&mdash;&mdash;结构调整步伐加快，产业水平稳步提升。坚持调结构、补短板、扩规模、增实力，全面加强产业建设。旅游转型迈出坚实步伐。澧水风貌带建设加快推进，特色街区、特色旅游风情镇等转型平台建设前期工作抓紧实施。旅游与文化、体育等融合发展取得新成效，《天门狐仙》和《魅力湘西》等旅游文化演艺业实现改造提质。户外旅游产品初现端倪，中德户外运动旅游联盟正式启动。自助游服务体系进一步完善，休闲度假、文化体验、康体疗养等新型旅游产品不断丰富。农业产业化发展规模不断扩大。制定农业产业提质升级&ldquo;523行动计划&rdquo;，以大鲵、蔬菜为发展重点调整优化农业结构。创建粮食高产示范片区17.8万亩，粮食总产达到63.6万吨。新增大鲵等畜禽水产养殖大户300户、蔬菜等优势产业规模种植基地5.5万亩、市级以上农业产业化龙头企业23家、农民专业合作社130家，扩大农村承包土地流转面积2.5万亩。园区经济和规模工业较快增长。完成工业园区基础设施建设投资3.3亿元，新增入园规模工业企业5家，园区规模工业增加值同比增长14%，占全部规模工业增加值的比重达到24 %。创新园区发展思路，实现市经济开发区与长沙经济技术开发区合作发展。大力推进传统工业企业技术改造，实现技改投资32亿元，同比增长25%；高新技术产业增加值达到3.56亿元，同比增长8%。开工兴建工业项目19个，完成投资6.56亿元。新增规模工业企业21家，规模工业增加值同比增长11.5%。服务业创新发展。创新金融服务平台，引进了华融湘江银行张家界分行，组建了张家界农商银行，完成了金融IC卡行业应用标准化改造。培育流通企业成长，新增限额以上商贸流通企业30家，实施了10个农贸市场标准化改造。改善商贸服务环境，启动4G网络建设，电子商务迅速发展；建成荷花国际机场新货运站，国际货运业务即将开通；农村&ldquo;新网工程&rdquo;建设取得明显成效。房地产业快速增长，开发投资同比增长116.8%，销售面积和销售金额同比分别增长21.2%和23.6%。非公有制经济健康发展，新增个体工商户3350户、私营企业1168家。

　　&mdash;&mdash;民生和社会建设扎实推进，人民群众生活质量持续改善。着力保障和改善民生，着力加强基本公共服务体系建设，着力构建和谐社会环境，切实把人民利益实现好、维护好、发展好。社会保障水平不断提升。千方百计保投入，高标准完成了31项省、市为民办实事工程。企业退休人员养老金、城乡低保、失业救济金发放标准和农村五保对象分散供养标准全面提高，城镇职工、居民医保年度支付总额进一步增加。实现五保对象在县乡医院、农村低保对象在乡镇医院住院个人零支付，免除农村产妇在县乡医疗机构分娩基本医疗费。新增城镇就业10176人、农村劳动力转移就业16669人，城镇零就业家庭保持&ldquo;动态清零&rdquo;。社会事业全面进步。积极推进义务教育均衡发展，治理在职教师有偿家教家养初见成效。高考成绩创历史新高。积极推进科技创新，获得国家和省级科技计划支持项目53个，发明专利授权量同比增长15%。慈利县荣获全国科技进步先进县。积极推进文体事业发展，群众文体活动更加活跃，广播电视覆盖率提高到93.5%，新增一批全国重点文物保护单位。积极推进医药卫生体制改革，基层医疗机构全部实行基本药物制度。人口自然增长率控制在6.87&permil;。积极推进各项事业发展，国防教育和后备力量建设不断加强，双拥工作、军民融合发展进一步深化，统计、审计、编制、气象、民族、宗教、残联、老龄、移民、对台、外事、侨务、档案、史志、无线电管理等各方面工作都取得了可喜的成绩。社会治理取得显著成效。强化安全生产责任，重点行业、重点领域隐患排查和整治全面落实，亿元GDP生产安全事故死亡人数稳定下降，安全生产跻身全省先进行列。食品药品、农产品质量安全监管体系全面建立。信访维稳力度加大，基层防控化解网络和应急处理机制进一步完善。&ldquo;平安创建&rdquo;深入推进，严打整治强劲有力，社会治安平稳可控，群众安全感、满意度测评位居全省前列。荣获&ldquo;全国法治城市创建活动先进城市&rdquo;、&ldquo;全国社会治安综合管理优秀地市&rdquo;，再次捧回了全国社会治理最高奖项&mdash;&mdash;&ldquo;长安杯&rdquo;。扶贫攻坚和新农村示范统筹推进。组织实施扶贫开发项目406个，建成了一大批改善贫困群众生产生活条件的基础工程，共减少贫困人口3万人。整合落实新农村示范片建设项目62个，扎实推进片区基础设施建设、主导产业发展和环境卫生整治，农村经济社会协调发展步伐不断加快。生态环境进一步改善。绿化工程有效推进，新增营造林10.5万亩，防治森林有害生物侵害18万亩，实行了公益林管理台账制度，全市森林覆盖率达到69.62%，保持全省领先水平。城乡环境同治力度加大，取缔城区99家企业燃煤锅炉和烟煤大灶，落实农村垃圾治理资金保障，淘汰了一批&ldquo;两高一低&rdquo;产能，建成了一批环保基础设施，全面完成省定节能减排任务。

　　一年来，在全力推进经济社会发展的同时，注重以改革创新的精神加强政府自身建设。大力推进依法行政，深入贯彻&ldquo;一规划两规定六办法&rdquo;，强化依法行政创新制度体系，着力加快政府职能转变，严格规范行政行为，让权力在阳光下运行。取消和调整了一批行政审批事项，审批时限进一步缩短。修订市政府工作规则，制定出台深入推进依法行政实施意见、重大行政决策程序规则、政府合同管理办法，发布行政执法指导案例，推进行政执法体制改革。自觉接受人大法律监督和工作监督、政协民主监督以及社会各界监督，认真办结了156件人大代表建议和172件政协委员提案。严格执行优化经济发展环境&ldquo;十条禁止性规定&rdquo;，落实改进作风&ldquo;十项措施&rdquo;，实行&ldquo;六个一律免职&rdquo;问责，压缩&ldquo;三公经费&rdquo;支出。

　　各位代表，在即将过去的一年里，我们奋力克服全局性、周期性、结构性、灾害性等多重困难，取得了来之不易的成绩。这是党中央、国务院，省委、省政府和市委正确领导的结果，是市人大、市政协以及社会各界大力支持的结果，是全市上下凝心聚力、真抓实干、攻坚克难的结果！在此，我代表市人民政府，向全市各族人民，向驻张部队、武警官兵和政法干警，向各民主党派、工商联、人民团体和各界人士，向所有关心支持张家界发展的朋友们，表示衷心的感谢！

　　各位代表，在看到成绩的同时，还必须清醒地看到存在的问题。一是全市经济发展的总量、质量、均量不高，与全面建成小康社会的目标要求还有很大差距。二是产业结构性矛盾仍然突出，以旅游业为主支撑发展的局面尚未得到根本性改观，旅游产业转型缓慢，工业短腿效应明显，经济整体抗风险能力非常脆弱。三是基础设施建设相对滞后，城市建设管理与人民群众的期盼和国内外知名旅游胜地要求还有不小的差距。四是资金、土地、人才等要素保障乏力，投资融资机制和平台建设亟需加强，财政实力严重不足。五是人民生活总体水平仍然较低，城乡居民收入不高，还有相当一部分群众处于贫困状态，扶贫攻坚的任务还相当艰巨。&ldquo;问题是目标与现实的差距&rdquo;，也就是&ldquo;旅游胜地梦&rdquo;、&ldquo;全面小康梦&rdquo;的目标与发展阶段、现实基础的差距，我们必须勇于直面这些问题，采取更加得力的举措解决这些问题。

　　二、2014年工作安排

　　2014年是全面建成小康社会的重要之年，是实施&ldquo;十二五&rdquo;规划的关键之年，也是我市实施&ldquo;提质张家界，打造升级版&rdquo;战略的攻坚之年。我们肩负着加快发展、加快转型的双重重担，任务十分艰巨。展望新的一年，机遇和挑战并存。世界经济仍处于深度调整期，国际环境充满复杂性和不确定性。我国已进入经济增长的换挡期、结构调整的阵痛期和刺激政策的消化期，改革发展仍然面临不少困难和挑战。我市作为后发地区的基本特征还十分明显，基础差、底子薄的基本市情没有根本改变，发展的内生动力严重不足。对此，我们必须保持清醒地认识，切实增强忧患意识。与此同时，我们更要看到我市面临的发展新机遇。党的十八届三中全会作出了全面深化改革若干重大问题的决定，必将释放更大的市场活力。国家武陵山片区区域发展与扶贫攻坚等重大机遇，正在不断转化为发展效应。省委、省政府实行全面建成小康社会分类指导，继续实施大湘西开发战略，不断加大对大湘西板块建设和发展的帮扶力度，为我市加快发展、加快转型带来了诸多利好。我市加快推进国内外知名旅游胜地建设，已经描绘了未来五年的发展蓝图，发展思路更加清晰、任务更加明确；一批重大项目的相继实施和重要基础工作的扎实开展，全市经济社会发展的动力将更加强劲。发展的进程没有坦途，发展的责任催人奋进。全市上下要清醒认识和把握发展形势，振奋精神，坚定信心，勇敢担起历史责任，全力以赴开创明年各项工作新局面。

　　2014年推进经济社会发展的总体要求是：以党的十八大和十八届二中三中全会精神为指导，认真贯彻落实中央和省委经济工作会议、市委六届五次六次全会的部署安排，紧紧围绕建设国内外知名旅游胜地和全面建成小康社会目标任务，以科学发展为主题，以加快转型为主线，以改革创新为动力，坚持&ldquo;稳中求进、好中求快&rdquo;总基调，落实&ldquo;三量齐升、分类指导&rdquo;总要求，实施&ldquo;提质张家界、打造升级版&rdquo;总战略，注重基础设施建设，加快转方式调结构，统筹城乡协调发展，加强基本公共服务，增进人民福祉，促进社会和谐，为早日实现&ldquo;旅游胜地梦&rdquo;和&ldquo;全面小康梦&rdquo;奠定坚实基础。

　　&mdash;&mdash;突出抓好项目建设。坚持把项目建设摆在更加重要的位置，把扩大投资作为更加关键的举措，强力开展&ldquo;项目建设突破年&rdquo;活动，着力实施一批打基础、利长远的基础设施项目，着力实施一批促转型、强实力的产业发展项目，着力实施一批排民忧、解民难的民生改善项目，掀起项目建设新高潮。

　　&mdash;&mdash;突出抓好产业发展。坚持&ldquo;产业兴市、产业强市&rdquo;，加快做大产业规模、优化产业结构、提升产业质量。实施&ldquo;调一进二提三&rdquo;发展方略，深入调整农业结构，大力推进工业跨越发展，全面提升服务业发展水平，加快旅游产业转型，促进房地产业可持续发展，着力构建旅游业为主导、新型工业为支撑、现代农业为基础、现代服务业为依托的具有张家界特色的现代产业体系，切实提高经济发展规模、质量和效益。

　　&mdash;&mdash;突出抓好民生改善。坚持民生优先，着力解决人民群众最关心、最直接、最现实的利益问题，千方百计增加城乡居民收入，加快发展社会事业，扎实推进扶贫攻坚，不断优化生态环境，认真解决群众就业、上学、看病、住房、出行、饮水等方面的实际困难，切实关注残疾人、五保户、低保户等特殊困难群体，推进基本公共服务均等化，让发展成果更多更公平惠及全体人民。

　　&mdash;&mdash;突出抓好改革创新。深入贯彻落实中央全面深化改革的决定，把改革创新贯穿于经济社会发展各个领域各个环节，以改革创新促进经济发展、转型升级、民生改善。着力深化旅游综合改革，积极推进行政体制、行政审批、政府机构、城管体制、投融资体制、医药卫生体制、事业单位、国有企业等各项改革，坚决破除影响发展、制约转型的体制机制弊端，在改革创新中激发发展活力，不断促进社会公平正义。

　　&mdash;&mdash;突出抓好作风转变。认真开展群众路线教育实践活动，坚决反对形式主义、官僚主义、享乐主义和奢靡之风。着力改进机关工作，强化制度约束，规范行政行为，优化政务服务，全面提高行政效能。厉行节约、艰苦奋斗，压缩行政成本，从张家界的实际出发解决张家界的实际问题，坚持勤俭办一切事情。

　　2014年全市经济社会发展的预期目标是：生产总值增长10%，其中一、二、三次产业增加值分别增长3.5%、11.6%、10.6%；景点接待旅游人次和旅游总收入分别增长5%和8%，过夜游客增长5%；规模工业增加值增长13%；固定资产投资增长20%；实际利用内、外资分别增长10%；社会消费品零售总额增长13%；进出口总额增长15%；财政总收入增长12%，城镇居民人均可支配收入和农民人均纯收入分别增长12%；居民消费价格涨幅控制在4%以内；城镇登记失业率控制在4%以内，人口自然增长率、安全生产、节能减排实现省定目标；社会事业全面进步，民主法制和精神文明建设进一步加强，社会大局更加和谐稳定。

　　落实明年经济社会发展的总体要求和目标任务，全市上下必须重点做好以下九个方面的工作：

　　（一）全力推进重大基础设施建设，确保投资持续快速增长。坚持以项目扩投资、以投资促项目，充分发挥投资拉动增长的关键作用，突出抓好以交通为重点的重大基础设施建设。加大项目推进力度。开工建设张桑、安慈高速公路，力争开工建设黔张常铁路，加快实施荷花国际机场新航站楼建设工程，大力推进东洋渡大桥、双门岛大桥、岩泊渡大桥、绕城西线，以及邢大、教温、杨通、零龙、刘洪、官瑞等干线公路建设，确保实现年度目标。继续夯实宜张、桑龙、张新高速公路等一批重点项目前期工作，力争早日落地建设。全面完成通讯网络、通畅公路、城乡电网等年度建设任务。大力突破要素瓶颈。深化投融资体制改革，优化金融生态环境，加大政银企合作对接力度，支持发展中小银行，规范发展小额贷款公司和融资性担保公司，力争金融贷款余额达到339亿元。积极控制和化解政府性债务风险，更好发挥经发投、交建投等融资平台作用。不断拓宽融资渠道，全面激活民间资本。推进土地利用总体规划修编，积极争取建设用地指标，加大闲置土地清理处置力度，依法依规破解征地拆迁难题。不断提高招商引资实效。进一步完善&ldquo;统分结合、归口管理&rdquo;的招商机制。加强招商引资基础工作，推进项目库和客户库建设，搞好项目开发和包装，提高招商项目质量。进一步创新招商方式，充分利用外商投资企业协会、经济促进会、商会等平台，广泛开展以商招商、联谊招商、小分队招商、点对点招商，争取在重大项目招商上实现新突破。

　　（二）加快旅游产业提质转型，着力推进旅游经济总量质量同步提升。坚持一手抓转型，一手抓营销，调结构，扩总量，稳定观光产品，发展休闲产品，丰富旅游产品体系，力促旅游市场实现恢复性增长。抓好转型项目，促进产业融合。着力抓好高星级酒店建设，开工兴建温德姆、家园国际、公园华邑、远大蓝色港湾等项目，兴建碧桂园、盛美达、华天城高星级酒店二期工程。着力抓好特色街区建设，启动南门口、仙人溪、武陵源城区等特色街区项目建设。着力抓好户外休闲产品建设，策划实施户外休闲项目，打造户外休闲基地。着力抓好自助游产品建设，加快完善自驾游标识系统，健全自助游服务体系。着力抓好红色旅游产品建设，增强红色旅游市场竞争力。加强景区基础设施建设，加快建设武陵源游客服务中心、锣鼓塔生态停车场、张家界大峡谷玻璃桥、旅游航道等项目，改造提升天子山索道，建成运营杨家界索道。推进相关产业建设，不断深化旅游与文化、体育、医疗、现代农业等融合发展，做大做强文化体验、体育赛事、康体疗养、休闲农业等产品产业。加快天门山先导区建设。深化营销创新，拓展客源市场。突出抓好&ldquo;五大营销&rdquo;。创新活动营销，充分调动市场主体积极性，精心筹办好第3届世界翼装飞行锦标赛、全国皮划艇激流回漩锦标赛、第4届中国自行车挑战赛等重大促销活动。创新客源地营销，巩固传统客源市场，开拓新兴客源市场，开展客源地企业、学校、协会、俱乐部等多元合作；组织好韩国、港澳台和东南亚等重要境外客源市场宣传促销，继续开拓北美市场，积极开发欧洲市场。创新网络营销，加强与乐途、携程等专业旅游网站合作，积极与FACEBOOK等社交网络平台合作，加快推进以张家界旅游官方网站为重点的&ldquo;智慧旅游&rdquo;系统建设。创新事件营销，建立激励机制，吸引创意策划人才，敏锐把握热点事件，制造旅游市场兴奋点。创新政策营销，进一步完善旅游营销奖励办法，鼓励开辟新的航线和专列，适时采取更加有效的市场刺激措施。推进旅游综合改革，强化旅游市场管理。创新旅游行政管理、旅游质量监督、旅游景区管理、旅游行业管理等体制，推进旅行社、旅游饭店、旅游客运等市场主体改革创新，切实增强行业自律能力和发展活力。加大《旅游法》贯彻实施力度，深化&ldquo;平安满意在张家界&rdquo;专项行动，扎实推进全国旅游服务标准化试点城市建设，大力整治和规范旅游市场秩序，严厉打击非法经营旅游业务、价格欺诈、欺客宰客、追客赶客等不良行为，优化旅游市场环境，不断提升旅游美誉度和满意度。加快现代服务业发展，促进消费结构升级。积极发展现代物流业，力争开通航空国际货运业务。改造提升商贸零售业，推进家乐福、中商广场等项目建设，加强综合批发市场、专业市场、城乡集贸市场建设。加快发展特色餐饮业，致力打造具有地域和民族特色的餐饮品牌。培育发展会展业，鼓励支持会展服务企业专业化、品牌化发展，扩大会展经济规模。大力发展信息服务业，启动4G网络商业运营，促进电子商务等新兴服务业态加快发展。

　　（三）加强城镇建设和管理，大力提高城镇化发展质量。科学有序、积极稳妥地推进新型城镇化，不断提升城镇建设和管理水平。切实把握城镇化发展方向。坚持城镇建设&ldquo;人本化&rdquo;，以人为本，推进以人为核心的城镇化，着力促进农业转移人口市民化，建设人民群众安居乐业的幸福城镇。坚持城镇建设&ldquo;特色化&rdquo;，既融入现代元素，又延续历史文脉，着力建设彰显张家界特色的现代建筑，建设具有历史记忆、地域特色、民族特点的魅力城镇。坚持城镇建设&ldquo;生态化&rdquo;，节约集约利用土地、水、能源等资源，把城镇放在大自然中，把绿水青山保留给城镇居民，建设布局优化、绿色低碳的生态城镇。坚持城镇建设&ldquo;产业化&rdquo;，推进产业兴城、产城融合，把城镇作为产业发展的重要载体，因地制宜发展城镇旅游业、城镇工业、城镇服务业，建设具有经济实力的富裕城镇。大力实施中心城区建设。围绕&ldquo;拓展新城两区、提质澧水两带&rdquo;，实施城区建设&ldquo;四大工程&rdquo;。实施&ldquo;道路建设工程&rdquo;，开工建设澧水两岸&ldquo;滨水环线&rdquo;，率先建设澧水南岸澧兰路、澧兰桥，力争三年全线贯通；启动武陵山大道、天问路、回龙路、机场第二通道和荷花大桥等建设项目。实施&ldquo;排污治污工程&rdquo;，开工建设东门溪排污设施，改造澧水沿线污水管网，实现三望坡、煤炭湾两个垃圾填埋场正常运行。实施&ldquo;市政设施工程&rdquo;，继续推进公厕、停车泊位、人行道板、垃圾收集站点等综合配套和改造提质建设。实施&ldquo;环境整治工程&rdquo;，以&ldquo;绿化、硬化、美化、净化&rdquo;为目标，加快市城区环境改造提质步伐。统筹推进县城和旅游风情镇建设。完成桑植县城老观潭组团建设工程，加快慈利县新城区项目实施，有序推进索溪峪、天子山、大坪、教字垭、江垭、溪口、洪家关、刘家坪等15个特色旅游风情镇建设。全面加强城市管理。改革创新城市管理体制，强化管理责任落实机制。改革城管执法体制，完善综合执法和相对集中行政处罚权制度。继续加大违法建设整治力度，坚决杜绝新的违法建设。深入推进市容市貌、环境卫生、交通秩序等综合治理，有效解决车辆乱停、渣土撒漏、噪声扰民、鞭炮燃放等突出问题。统筹推进国家森林城市、园林城市、环保模范城市、卫生城市、交通管理模范城市和省级文明城市&ldquo;六城同创&rdquo;工程，不断提升城市品位。切实加强城市社区建设，深入开展&ldquo;三联四建&rdquo;活动。不断完善城镇发展规划。完成城市总体规划实施评估，批准实施城乡一体化等专项规划和沙堤片区控制性详规，加快编制村镇规划。深化法定图则管理，切实规范城镇建设秩序。

　　（四）加快发展现代农业，扎实推进新农村建设。全面建成小康社会最艰巨最繁重的任务在农村。要以发展现代农业为重点，全面促进&ldquo;三农&rdquo;发展，切实加强新农村建设。加快农业产业化进程。全面实施农业产业提质升级&ldquo;523&rdquo;行动计划，着力壮大大鲵、蔬菜两大产业，扩大大鲵养殖规模30万尾，发展商品蔬菜基地6万亩。稳定粮油生产，确保粮食安全。巩固提高畜牧、水果、烟叶、中药材产业，稳定发展茶叶、油茶、花卉苗木、休闲农业产业，打造富硒农产品品牌。支持农业产业化龙头企业加快发展，扩大农产品加工转化规模，提高农产品商品化水平。加快农村基础设施建设。完成农村土地连片综合整治5800公顷，力争补充耕地5000亩。改造和完善200个村的供电网络，开工兴建各类水利工程8760处，修建和改造农村公路240公里，完成5000个农户清洁能源建设任务，新增12万农村安全饮水人口。加快农业农村服务体系建设。健全农技、农机、农资、气象、信息等服务网络，构建农业社会化服务新机制。新建农民专业合作社100家，力争新增土地流转面积3万亩。强化农产品质量安全监督管理，加大动植物病虫害防治力度。抓好粮食危仓老库改造，加快&ldquo;新网工程&rdquo;项目建设，完善农产品市场体系，切实搞活农产品流通。加快扶贫攻坚和新农村示范建设。坚持以&ldquo;产业扶贫&rdquo;为重点，以桑植县为主战场，整合资金，连片推进，全面落实各项建设扶贫任务，力争实现减少3万贫困人口目标；加大&ldquo;百城千镇万村&rdquo;新农村示范片的创建力度，确保完成年度建设任务。扎实推进农村&ldquo;四培四带&rdquo;活动，切实加强农村基层组织建设。

　　（五）培育壮大工业经济，力促新型工业快速成长。推动全市经济新一轮快速发展，做大做强工业是关键。要坚持绿色、低碳、高效的发展理念，坚持&ldquo;抓大扶小&rdquo;的基本发展方略，着力培育大园区、大项目、大企业，着力扶持小微企业发展，不断增强工业经济发展实力。加快推进园区建设。加强园区招商，力争引进一批&ldquo;大强高优&rdquo;工业企业落户园区。依托国家级品牌优势，深化与长沙经济技术开发区合作。着力完善园区基础设施配套，实现园区建设投资4亿元。完成神舟药业中药饮片生产线建设和华新水泥技改工程，开工建设锦华药业、恒久高新、立方石业等项目，力争新增入园企业20家、园区工业增加值增长20％。做大做强特色工业。大力发展生物医药、旅游商品、食品加工、新型材料、清洁能源工业。重点抓好五倍子、杜仲、黄柏等资源的开发利用和精深加工。深度开发大鲵、葛根、茅岩莓等特色资源，规划建设旅游商品产业园。推进食品工业园项目落地建设。支持恒亮矿业、永兴玻璃等企业加快发展，促进大理石资源深度开发。争取岩泊渡、淋溪河电站开工建设，加快桑植、慈利页岩气开发。力争规模工业企业达到160家，产值过亿元企业达到30家，税额过千万元企业达到12家。鼓励扶持小微企业发展。全面落实促进小微企业发展各项扶持政策，筹措小微企业发展引导资金，推动小微企业做优做强，力争新增小微企业500户以上。引导促进企业清洁生产。大力改造传统生产工艺，推进循环工业发展。引导和支持&ldquo;清洁生产企业&rdquo;创建活动，力争5至10家工业企业通过清洁生产审核。大力推进企业减排任务落实，突出抓好企业脱硫脱硝治理、污染排放设施建设、实心粘土砖生产行业整治，促进工业&ldquo;两型&rdquo;发展。着力推动企业科技创新。加快以企业为主体的自主创新平台建设，积极构建产学研一体化科技创新体系，培育一批拥有自主知识产权、适应规模化生产、具有一定品牌效应的技术创新示范企业。加快推动&ldquo;两化&rdquo;融合工程，促进信息技术与产品研发、生产、经营、管理四大环节有机融合，力争企业技术改造投资增长20％以上。

　　（六）巩固壮大文化品牌优势，大力提升文化软实力。文化软实力是综合竞争实力的重要体现。要把文化建设摆在更加突出的位置，加快发展文化产业和文化事业，丰富文化旅游产品，强化文化服务功能，不断满足广大市民和游客的文化需求。建立健全以文化旅游为重点的文化市场体系。培育文化市场主体，引导文化企业规模化、集约化、专业化发展，支持有条件的文化企业上市融资。打造文化旅游品牌，支持《天门狐仙》、《魅力湘西》进一步做优做强，扩大品牌的知名度和影响力。抓好文化街区、文化广场建设，促进演艺、娱乐、网络、艺术品、出版物市场以及印刷复制业快速健康发展。扶持新型文化业态发展，提高文化市场多元化供给能力。加快完善现代公共文化服务。加强公共文化基础设施建设，力争武陵山博物馆落户张家界，实施张家界图书城、市图书馆、澧水风貌带&ldquo;文化长廊&rdquo;等文化项目工程，促进贺龙国际体育中心复工兴建，实现市博物馆地质馆开馆运行。加强公共文化服务工程建设，启动广播电视&ldquo;户户通&rdquo;工程。加强群众文化活动场所建设，继续开展好送戏送电影下乡活动，扩大文化惠民活动覆盖面。加强张家界历史文化基础性研究，充分挖掘民族和地域文化内涵。认真做好桑植民歌、大庸阳戏等非物质文化遗产资源的保护、开发和利用工作，力争进入国家武陵山区文化生态保护实验区。加大文化市场管理力度。坚持正确舆论导向，注重新兴媒体的运用和管理，形成正面引导和依法管理相结合的网络舆论工作格局。强化&ldquo;扫黄打非&rdquo;，抵制低俗、庸俗、媚俗文化活动，净化文化市场。深入推进精神文明创建活动。支持举办市民学校和&ldquo;道德讲堂&rdquo;，推进社会主义核心价值观教育进机关、进学校、进企业、进村庄、进社区，大力开展文明城市、文明乡镇、文明社区、文明家庭创建活动，不断提高公民思想道德素质，树立良好社会风尚。

（七）切实保障和改善民生，统筹发展社会事业。采取更加有力的措施，努力解决好人民群众生产生活中的实际困难和问题，加快完善基本公共服务体系，着力创建良好社会环境，不断提高人民群众的&ldquo;幸福指数&rdquo;。进一步提高社会保障水平。统筹城乡就业，力争城镇新增就业9000人、失业人员再就业3000人、农村劳动力转移就业1.5万人。继续扩大社会保险覆盖面，拓展城乡养老保险覆盖范围，实现城乡低保应保尽保、医疗救助&ldquo;一站式&rdquo;结算全覆盖。建设各类保障性住房5000套以上，扎实推进农村危房改造工程，确保低收入群体居住困难不断得到解决。进一步优先发展教育事业。统筹抓好学前教育、义务教育、高中阶段教育，加快发展现代职业教育，支持高等学校做大做强。大力实施合格学校、薄弱学校改造等工程建设，不断改善办学条件。力争张家界一中扩建、张家界旅游学校等项目落地开工，加快推进市技工学校、张家界航院整体搬迁等项目建设。鼓励和规范社会力量办学，促进民办教育健康发展。合理配置城乡教育资源，着力解决&ldquo;大额班&rdquo;问题。抓好农民工子女、农村留守儿童、残疾儿童受教育问题，促进教育公平。切实维护校园安全，确保校车安全运行。进一步改善公共卫生服务。推进新农合制度由扩量向提质转变，巩固完善基层医疗机构运行新机制，提高公共卫生服务均等化水平。积极推进县级公立医院综合改革，合理整合市城区医疗卫生资源。切实加强疾病预防控制，加强医疗卫生监督。加大对医疗市场、卫生行风、非法行医等专项整治力度，严厉整治医药购销领域的不正之风，杜绝&ldquo;大处方&rdquo;及收受回扣、红包现象。加快推进市人民医院新建项目。着力提高人口计生工作水平。进一步促进科技创新。抓好国家和省级科技项目的申报和建设，力推大鲵、杜仲、五倍子、黄柏、葛根、碳酸钙等重大科研攻关项目取得实质性进展。积极推进科技成果孵化平台建设，组建张家界市大鲵产业技术创新战略联盟。加快发展高新技术产业，力争高新技术产业增加值增长20%以上。抓好科普、知识产权和防灾减灾工作。统筹推进各项社会事业发展。认真开展好第三次全国经济普查，扎实推进民政、民族、宗教、人民防空、国防后备力量建设、无线电管理以及移民、妇女、儿童、老龄、残联、普法等各项工作。充分发挥外事、侨务工作作用，加强对外友好平台建设，促进旅游国际化。进一步加强和创新社会治理。全面落实安全生产责任制，认真抓好安全生产大检查和安全隐患大排查，强化交通、消防、矿山等重点领域安全整治，严防重特大安全责任事故发生。大力实施&ldquo;质量强市&rdquo;战略，加强质量技术监督。积极稳妥推进食品药品监督体制改革，抓好食品、药品&ldquo;放心工程&rdquo;。加强市场价格调控管理，确保价格总水平基本稳定。深入创建&ldquo;平安张家界&rdquo;，健全完善应急联动机制和快速反应机制，加快推进&ldquo;天网工程&rdquo;，提高社会治安动态打防管控能力。加强和改进信访、综治维稳基础工作，开通网上信访，落实重大工程项目风险评估制度，完善维护群众权益工作机制，努力从源头上化解社会矛盾。进一步实施为民办实事工程。在全面落实省定实事项目的同时，市人民政府再兴办扶植培训技术工人、扶助失独家庭、补助精神病患者医疗费用、实施残疾人家庭无障碍改造等实事。

　　（八）加大生态环境保护力度，全面建设美好的绿色家园。保护生态环境就是保护生产力，改善生态环境就是发展生产力。生态环境是张家界经济社会发展的生命线。要坚持&ldquo;严格保护、有序开发&rdquo;，走出一条&ldquo;在保护中发展、在发展中保护&rdquo;的生态文明之路。切实加强世界自然遗产保护。严格执行《湖南省武陵源世界自然遗产保护条例》，加强保护世界自然遗产和地质遗迹各项工作，突出抓好松材线虫病、森林火灾和地质灾害的监测和防治，启动实施武陵源核心景区综合整治工程和移民搬迁工程。突出抓好环境整治。全面实施环境整治&ldquo;六大专项行动&rdquo;，突出治理扬尘、尾气，依法取缔城市周边砖厂和采石场，开展&ldquo;黄标车&rdquo;治理，启动景区城区车辆油改气工程。加强对重点行业水污染防治，确保奥威科技、城乡医院污水废水排放达标。深入推进城乡环境同治，统筹实施垃圾治理、畜禽养殖污染治理、噪声污染整治。扎实推进&ldquo;绿色张家界&rdquo;建设。大力开展植树造林，新增人工造林8万亩、封山育林18万亩。全面倡导绿色消费，大力弘扬绿色文化。着力发展生态环保产业，努力打造绿色产业品牌。加快推进澧水中上游综合治理、农村环境连片综合治理等生态环保项目建设。全面完成节能减排任务。突出抓好&ldquo;两型社会&rdquo;示范工程和示范单位创建活动，重点支持省级循环经济示范项目建设，完成桑梓电厂、南方水泥等重点企业环保提质升级工程。积极争取上级支持，在我市开展全省、全国生态效益补偿试点，建立健全生态效益补偿机制。

　　（九）切实加强政府自身建设，努力建设人民满意的政府。加快推进工作作风、政府职能、行政方式&ldquo;三个转变&rdquo;，不断提高政府公信力和执行力，全面构建法治政府和服务型政府。要进一步转变工作作风，树立良好形象。坚决反对形式主义，下决心精简会议、文件，改进会风、文风，真正把功夫下到察实情、出实招、办实事、求实效上，树立求真务实的良好形象。坚决反对官僚主义，接地气、通下情，下大气力解决门难进、脸难看、事难办，敷衍塞责、效率低下、小事拖大、大事拖炸，不作为、乱作为、缓作为，权力部门化、部门利益化等问题，树立勤政为民的良好形象。坚决反对享乐主义，努力克服沉迷玩乐、不讲工作只讲待遇、不追求奉献只追求享受的不良倾向，激励各级干部不怕吃苦、不怕困难，勇于担责、勇于干事，树立艰苦创业的良好形象。坚决反对奢靡之风，努力克服虚荣摆阔、挥霍浪费倾向，切实加强廉政教育和廉政制度建设，强化纪律要求，严格约束干部行为，管住嘴、管住手、管住腿，树立廉洁奉公的良好形象。要进一步转变政府职能，强化政务服务。坚持&ldquo;管制做减法、服务做加法&rdquo;，切实把政府工作重点转到创造良好发展环境、提供优质公共服务、维护社会公平正义上来。深入推进行政审批制度改革，精简审批程序，提高限时办结率和满意度。加强电子政务和电子监察系统建设。强化服务意识，着力为市民、游客、企业提供优质高效服务。大力优化经济发展环境，营造重商、亲商、安商、富商良好氛围，严厉查处和纠正损害群众利益和发展环境的人和事，严厉打击强行参工参运、阻工霸工、敲诈勒索等破坏发展环境的不法行为。完善绩效考核办法，严肃考核结果运用，兑现行政问责制度，全面提高行政效能。要进一步转变行政方式，推进依法行政。切实加强民主法制，建设&ldquo;法治张家界&rdquo;，努力提高运用法治思维和法治方式深化改革、推动发展、化解矛盾、维护稳定的能力。自觉接受人大及其常委会的法律监督、工作监督和政协的民主监督。切实抓好重大行政决策程序规则贯彻落实，努力提高行政决策科学化、民主化、法治化水平。切实规范行政执法行为，全面落实行政程序规定和政府服务规定，严格实施规范行政裁量权办法，努力提高行政执法水平。

　　各位代表， 回顾2013年，我们意气风发，奋力前行；展望2014年，我们责任重大，信心满怀。让我们紧密团结在以习近平同志为总书记的党中央周围，在省委、省政府和市委的坚强领导下，同心同德，锐意进取，为早日实现&ldquo;旅游胜地梦&rdquo;、&ldquo;全面小康梦&rdquo;而努力奋斗！
